# Endocytic uptake of monomeric amyloid-β peptides is clathrin- and dynamin-independent and results in selective accumulation of Aβ(1–42) compared to Aβ(1–40)

**DOI:** 10.1038/s41598-017-02227-9

**Published:** 2017-05-17

**Authors:** Emelie Wesén, Gavin D. M. Jeffries, Maria Matson Dzebo, Elin K. Esbjörner

**Affiliations:** 1 Division of Chemical Biology, Department of Biology and Biological Engineering, Chalmers University of Technology, Kemivägen 10, 412 96 Gothenburg, Sweden; 2 Division of Chemistry and Biochemistry, Department of Chemistry and Chemical Engineering, Chalmers University of Technology, Kemivägen 10, 412 96 Gothenburg, Sweden

## Abstract

Intraneuronal accumulation of amyloid-β (Aβ) peptides represent an early pathological feature in Alzheimer’s disease. We have therefore utilized flow cytometry and confocal microscopy in combination with endocytosis inhibition to explore the internalisation efficiency and uptake mechanisms of Aβ(1–40) and Aβ(1–42) monomers in cultured SH-SY5Y cells. We find that both variants are constitutively internalised via endocytosis and that their uptake is proportional to cellular endocytic rate. Moreover, SH-SY5Y cells internalise consistently twice the amount of Aβ(1–42) compared to Aβ(1–40); an imaging-based quantification showed that cells treated with 1 µM peptide for 8 h contained 800,000 peptides of Aβ(1–42) and 400,000 of Aβ(1–40). Both variants co-localised to >90% with lysosomes or other acidic compartments. Dynasore and chlorpromazine endocytosis inhibitors were both found to reduce uptake, particularly of Aβ(1–42). Overexpression of the C-terminal of the clathrin-binding domain of AP180, dynamin2 K44A, or Arf6 Q67L did however not reduce uptake of the Aβ variants. By contrast, perturbation of actin polymerisation and inhibition of macropinocytosis reduced Aβ(1–40) and Aβ(1–42) uptake considerably. This study clarifies mechanisms of Aβ(1–40) and Aβ(1–42) uptake, pinpoints differences between the two variants and highlights a common and putative role of macropinocytosis in the early accumulation of intraneuronal Aβ in AD.

## Introduction

The pathology of several severe and debilitating neurodegenerative disorders are characterized by the misfolding and aberrant self-assembly of specific proteins into extracellular deposits or intracellular inclusions^1^. Alzheimer’s disease (AD), the most prevalent form of adult dementia, is associated with the formation of extracellular plaque deposits, whose main proteinaceous constituent is the amyloid-β (Aβ) peptide^2, 3^. Aβ has therefore been suggested as a causative agent of AD pathology^4^. Many familial forms of AD are strongly associated with mutations that increase the aggregation propensity of Aβ or alter its production, processing and clearance^5–9^. A recent *in vitro* human cell model of AD suggests that Aβ plaque formation precedes and even drives the development of neurofibrillary tau tangles^10^, a second pathological lesion in AD^11^, but the prevalence of extracellular Aβ plaques in the human brain does not correlate to the severeness of cognitive decline^12^. Evidence from human brain samples and transgenic mice suggests that plaque formation is preceded by an intraneuronal build-up of Aβ^13–15^. This conspicuously occurs alongside an emergence of morphological aberrations to vesicular organelles of the endolysosomal system^16^.

Aβ peptides are generated in neurons by sequential proteolytic cleavage of the membrane-bound amyloid precursor protein (APP) by two aspartyl proteases: β-secretase and γ-secretase^17^. Most of the APP cleavage typically results in the 40 residue variant Aβ(1–40) (80–90%) and thereafter the 42 residue variant Aβ(1–42) (5–10%)^18^ which is neurotoxic^19^, predominant in extracellular plaques^20^ and selectively accumulated in intraneuronal locations^15^. Aβ production occurs in acidic vesicular organelles and contributes both to Aβ secretion and to the direct accumulation of Aβ variants within neurons^21^; recent evidence suggests that the subcellular location of presenilin2 sensitively regulates γ-secretase activity to specific endolysosomal compartments and hence the balance between intracellular accumulation and secretion^22^. In addition, both *in vitro* and *in vivo* studies show that extracellular Aβ can be re-internalised and concentrated in neurons^23, 24^. We have recently shown that this leads to selective enhancement of intracellular Aβ(1–42) aggregation compared to that of intracellular Aβ(1–40)^25^. Herein we explore the mechanisms by which neuronal cells internalise these two most common Aβ variants. This is important from the perspective of how re-uptake of Aβ may contribute to the molecular pathology of AD.

We have studied the cellular uptake of fluorescently labelled soluble Aβ(1–40) and Aβ(1–42) primarily in human SH-SY5Y neuroblastoma, using flow cytometry and confocal microscopy, to quantitatively address their uptake characteristics. We have both determined their absolute uptake and compared their relative internalisation rates. We have also perturbed different endocytic paths in SH-SY5Y cells using pharmacological inhibitors and genetic approaches to evaluate the pathways by which these two peptides enter cells. Our data conclusively show that soluble Aβ(1–40) and Aβ(1–42) use endocytosis to enter cells. Aβ(1–42) is taken up two times more efficient than the two residue shorter Aβ(1–40) and we find that although some differences exist in their responsiveness to endocytic inhibitors, both peptides are taken up via predominantly clathrin- and dynamin-independent mechanisms that are consistent with macropinocytosis.

## Results

### Aβ(1–40) and Aβ(1–42) are constitutively internalised into SH-SY5Y cells but with different efficiencies

**Figure 1:**
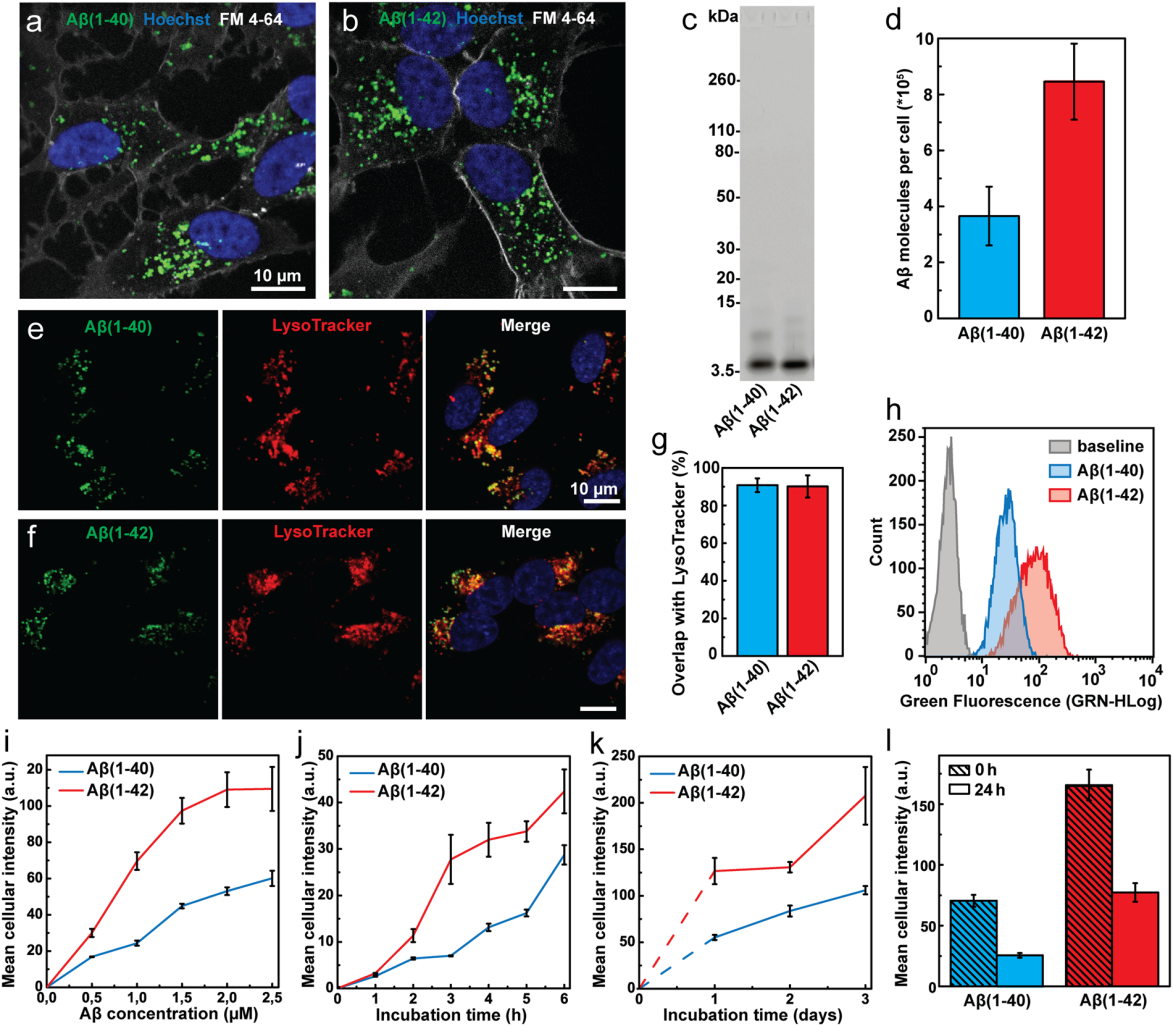
Imaging and quantification of the cellular uptake of Aβ(1–40) and Aβ(1–42) in SH-SY5Y cells. (**a**,**b**) Confocal microscopy images showing uptake of HF488-labelled Aβ(1–40) (**a**) and Aβ(1–42) (**b**) (green) after 24 h incubation at 500 nM peptide concentration. The cell nuclei (blue, Hoechst 33342) and outer cell membrane (grey, FM 4–64) were stained immediately prior to imaging. (**c**) SDS-PAGE of Aβ(1–40) and Aβ(1–42) solutions. The major band corresponds to HF488-labelled Aβ(1–40) and Aβ(1–42) monomers (4.7 and 4.9 kDa respectively). (**d**) Confocal imaging based quantification of the cellular uptake of Aβ(1–40) and Aβ(1–42) following incubation with 1 µM Aβ(1–40) or Aβ(1–42) for 8 h. The average uptake of Aβ molecules per cell was 3.7e^5^ ± 1.1e^5^ for Aβ(1–40) and 8.5e^5^ ± 1.6e^5^ for Aβ(1–42). The data is presented as mean ± SD from the analysis of 16 images from two separate culture dishes. Each image showed on average 25 cells. (**e**–**g**) Co-localisation analysis of confocal microscopy images (**e**,**f**) showing Aβ signal overlap with LysoTracker Deep Red. (**g**) Mander’s coefficient ± SD for the overlap of Aβ with Lysotracker Deep Red was calculated by analysis of 21 images with ~5 cells in each image (from three separate samples). The cells were incubated for 24 h, washed and incubated for an additional 5 h prior to imaging. (**h**) Flow cytometry histograms showing the cellular uptake of Aβ(1–40) and Aβ(1–42) following incubation with 1 μM peptide for 5 h. The baseline, representing cellular autofluorescence, is shown for comparison. (**i**) Uptake of Aβ(1–40) and Aβ(1–42) as function of peptide concentration following 5 h of incubation. (**j**,**k**) Uptake of Aβ(1–40) and Aβ(1–42) as function of incubation time at a peptide concentration of 500 nM. (**l**) Clearance of intracellular Aβ(1–40) and Aβ(1–42). Cells were loaded by incubation with 500 nM peptide for 24 hours (“0 h”), washed and incubated for an additional 24 h (“24 h”). The data has been corrected for cell division. Uptake in (**i**–**l**) is reported as mean cellular fluorescence intensity ± SD of the total number of gated live cells in three replicates. Data were corrected for baseline contributions by subtracting the mean cellular autofluorescence.

In order to quantitatively determine the extent of cellular uptake of Aβ(1–40) relative to Aβ(1–42), we exposed human neuroblastoma SH-SY5Y cells to low concentration (nanomolar to low micromolar) solutions of Hilyte Fluor™ 488 (HF488)-labelled variants of these two peptides (hereafter denoted as Aβ(1–40) or Aβ(1–42)). The peptide solutions were pre-treated by dissolving lyophilised peptide powder in hexafluoro-2-propanol, which was subsequently removed by rotary evaporation, a procedure that is known to dissolve any pre-formed Aβ aggregates by disruption of β-sheet structures^26^. After this pre-treatment, the peptide solutions, diluted to 1 µM concentration, were analysed by SDS-PAGE, showing that the majority of the peptides run as an expected ~5 kDa labelled monomer (Fig. 1c). Faint bands corresponding to dimers and trimers could also be observed, but no higher order species. Quantitative analysis of the band intensities in each lane showed that the monomer fraction was 84% for Aβ(1–40) and 86% for Aβ(1–42). This result strongly suggests that there are no inherent differences in the preparations of the peptides at start of the experiments. Furthermore, fluorescence emission spectra recorded on equimolar concentrations of the Aβ(1–40) and Aβ(1–42) solutions show that the two labelled peptides have the same intrinsic intensity (see Supplementary Fig. S1). This finding is consistent with the total band intensities on the gel which were 2300 a.u. for Aβ(1–40) and 2500 a.u. for Aβ(1–42).

Confocal laser scanning microscopy images of cells treated with fluorescently labelled Aβ(1–40) (Fig. 1a) or Aβ(1–42) (Fig. 1b) for 24 h, show that both variants are taken up and localized into distinct puncta with predominately perinuclear localisation, consistent with accumulation in acidic compartments as reported previously^23, 25, 27^. We next determined the fraction of Aβ in the lysosomes (Fig. 1e–g). Cells were incubated with 500 nM of either Aβ(1–40) (Fig. 1e) or Aβ(1–42) (Fig. 1f) for 24 h, washed and thereafter incubated for an additional 5 h to avoid sampling transient location of peptides in organelles upstream of the lysosome. Co-localisation analysis of the recorded images with JACoP plug-in to ImageJ^28^ show that 90.8 ± 3.6% of Aβ(1–40) and 90.1 ± 5.9% of Aβ(1–42) colocalise with Lysotracker Deep Red stained compartments (Fig. 1g). This result shows that there is no difference between the two peptides with respect to their final destination within cells. Further, the images in Fig. 1a,b show that neither Aβ(1–40) nor Aβ(1–42) associate with the cellular membrane and that all cell-associated fluorescence which remains after mild washing with 1x serum-free media, emanates from within the cells. This demonstrates that the total cellular fluorescence represents intracellular peptide only. This is important for the experiments in this study as it validates the use mean cellular fluorescence intensity, from confocal laser scanning microscopy as well as flow cytometry, as a measure of uptake in a population of Aβ-treated cells. Next, we determined the number of internalised Aβ peptides in each cell by quantitative confocal microscopy analysis and comparison of pixel intensities against a standard curve recorded on *in vitro* solutions with known peptide concentrations (see Supplementary Fig. S2) as described in the Methods section. We find that cells that had been incubated with 1 µM solutions of Aβ(1–40) or Aβ(1–42) for a total of 8 h, internalised respectively on the order of 400,000 and 800,000 molecules per cell (Fig. 1d). To set this result in perspective we also re-calculated these numbers into intravesicular Aβ concentrations under the assumption of a total lysosomal load of 10 μm^3^ per cell^29^. This yields a concentration of ~60 µM Aβ(1–40) and ~140 µM Aβ(1–42) and suggests that cellular uptake can lead to a 100-fold concentrating effect, consistent with a previous suggestion by Hu *et al*.^23^. To further explore the difference in uptake we used flow cytometry to examine cells treated with either Aβ(1–40) or Aβ(1–42) for different incubation periods and with different peptide concentrations, using the total cellular intensity as a measure of the relative amount of internalized peptide (Fig. 1h–k). Only cells that were gated as live by the forward scatter/side scatter (FSC/SSC) (see Supplementary Figs S3 and S4) were counted and included in the analysis. Figure 1h shows representative flow cytometry histograms of the distribution of the mean cellular fluorescence in samples that had been exposed to 1 µM of Aβ(1–40) or Aβ(1–42) for a period of 5 h prior to analysis. The results show that the SH-SY5Y cells internalize the two Aβ variants in a concentration-dependent manner (Fig. 1i) and confirms our above observation that the relative amount of internalized Aβ(1–42) is approximately twice as high as the amount of Aβ(1–40); this result was valid across the entire range of tested concentrations (500 nM to 2.5 µM). We thereafter monitored the uptake of 500 nM Aβ(1–40) and Aβ(1–42) as a function of time; Fig. 1j shows results over the first 6 h of incubation and Fig. 1k shows the uptake over 3 days. It is noteworthy that the intracellular concentration of neither Aβ(1–40) nor Aβ(1–42) appears to saturate under the experimental conditions used in our study. We also measured the cellular capacity to clear Aβ(1–40) and Aβ(1–42) (Fig. 1l). Based on intracellular fluorescence intensity, when no more peptide was supplied to the cells, approximately 60% of the Aβ peptides were cleared after 24 h. No major differences in clearance were found between the two peptide variants.

### Cellular uptake of Aβ(1–40) and Aβ(1–42) occurs exclusively via endocytosis

**Figure 2:**
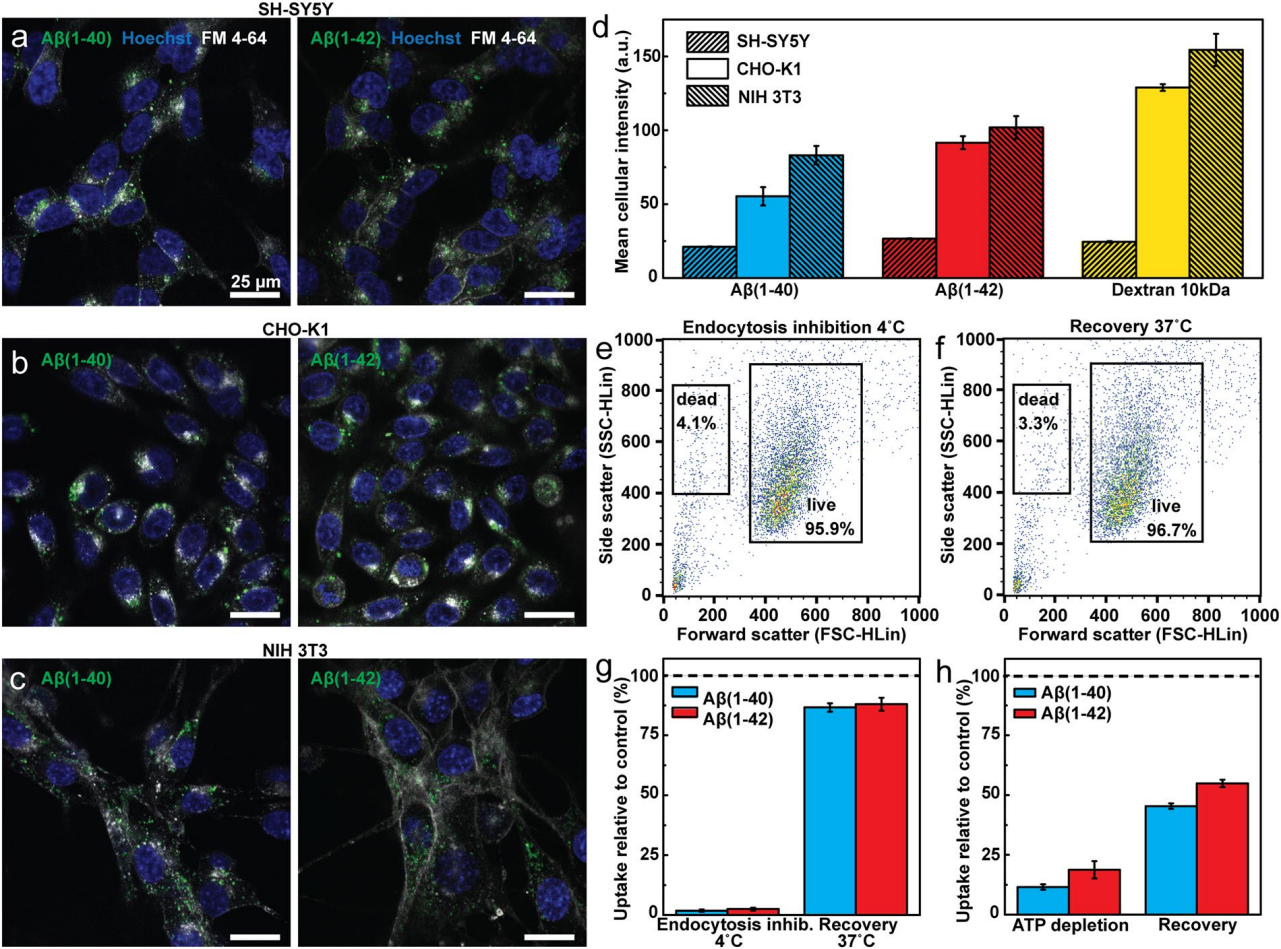
Endocytic uptake of Aβ(1–40) and Aβ(1–42) in different cell lines. (**a**–**c**) Confocal fluorescence microscopy images of live cells. ((**a**) SH-SY5Y, (**b**) CHO-K1 and (**c**) NIH 3T3) incubated with HF488-labelled Aβ(1–40) (left panels) or Aβ(1–42) (right panels) (green) for 1 h with a peptide concentration of 1 µM. Cell nuclei (blue) were stained with Hoechst 33342 (5 µg/ml) and the cell membrane and endosomes (grey) were stained with FM 4–64 (5 µg/ml) 10 min prior to imaging. (**d**) Cellular uptake of HF488-labelled Aβ(1–40), Aβ(1–42) and AF488-labelled dextran 10 kDa after 1 h incubation with respectively 1 µM Aβ(1–40), 1 µM Aβ(1–42) and 125 µg/ml dextran 10 kDa. (**e**,**f**) Scatter plots of the cell forward and side scatter (FSC/SCC) showing the fraction of the total number of counted cells gated within the live and dead gates after 2 h incubation at 4 °C (**e**) and after 2 h subsequent recovery at 37 °C (**f**). (**g**) Uptake of Aβ(1–40) and Aβ(1–42) in cells kept at 4 °C. The left bars show reduction in uptake relative to control (37 °C) upon 2 h incubation with 500 nM peptide and the right bars show uptake in cells that after 2 h incubation at 4 °C were allowed to recover, in presence of peptide for 2 h at 37 °C. (**h**) Uptake of Aβ(1–40) and Aβ(1–42) following ATP depletion with 10 mM 2-deoxy-D-glucose and 10 mM sodium azide in PBS. The cells were pre-treated with the ATP depletion solution for 2 h before addition of 1 μM Aβ(1–40) or Aβ(1–42) for an additional hour and cell uptake is reported relative to untreated control (left bars). The right bars represent uptake in cells that were allowed to recover in ATP-containing media for 2 h following depletion. The uptake is reported as mean cellular fluorescence intensity ± SD of the total number of gated live cells for three replicate samples (n = 3). All flow cytometry data were corrected for baseline contributions by subtracting the cellular autofluorescence.

We next tested whether the quantitative difference in uptake of Aβ(1–40) and Aβ(1–42) was cell line specific by comparing the uptake in SH-SY5Y cells to Aβ uptake in epithelial-like CHO-K1 cells and NIH 3T3 fibroblasts. The cells were incubated with 1 µM of Aβ(1–40) or Aβ(1–42) for 1 h. All cell types internalised the peptides into distinct puncta (SH-SY5Y in Fig. 2a, CHO-K1 in Fig. 2b and NIH 3T3 in Fig. 2c), indicative of uptake into endocytic compartments. These images further corroborate our above finding that Aβ does not associate to any measurable extent to the plasma membrane. Next, we quantified the relative Aβ uptake in the different cell lines using flow cytometry. This revealed that CHO-K1 and NIH 3T3 cells internalise both Aβ(1–40) and Aβ(1–42) with considerably higher efficiency compared to the SH-SY5Y cells (Fig. 2d). We used a fluorescent 10 kDa dextran as general fluid phase endocytosis marker^30^, to estimate the overall cellular endocytic capacity of the three cell lines. We found a qualitative correlation between Aβ uptake and the uptake of the 10 kDa dextran, strongly indicating that endocytosis is the major contributor to Aβ(1–40) and Aβ(1–42) internalisation in all studied cell types.

Focusing on SH-SY5Y cells, we next quantified the uptake of Aβ(1–40) or Aβ(1–42) in cells kept at 4 °C, as this extensively slow or halt all endocytic mechanisms^31^. After 2 h the cells were washed and thereafter immediately analysed by flow cytometry; Fig. 2g shows that uptake of both variants was reduced by more than 95% relative to untreated control, supporting the view that uptake of Aβ occurs exclusively via endocytosis, at least if the peptides are applied in their monomeric and soluble forms as was tested here. In addition, when the cells were allowed to recover from the 4 °C treatment they resumed ~80% of the uptake in the control samples, showing that the low temperature treatment induced a transient block in uptake. Furthermore, >95% of the low-temperature incubated and recovered cells were found within the live gate of the flow cytometry FSC/SSC dot-plots (Fig. 2e,f).

Low temperature incubation efficiently inhibits endocytosis, but it also perturbs the fluidity of the plasma membrane^32^ which could potentially influence the interactions between Aβ and the cell surface. Therefore we also measured Aβ(1–40) and Aβ(1–42) uptake in cells which had been depleted of ATP in order to disrupt all energy-dependent uptake paths^33^. This reduced the uptake of both Aβ(1–40) and Aβ(1–42) by more than 80% relative to untreated control (Fig. 2h), but ATP depletion also influenced cell viability. At the conditions used, 25% of the cells detected were gated as dead and thus excluded from the analysis (see Supplementary Fig. S5). Yet, we found that if we transferred the ATP-depleted cells back into normal serum-free medium for 2 h at 37 °C, they resumed their uptake relative to the untreated control (~50%, Fig. 2h), suggesting that the surviving cells were able to at least partially recover. Taken together, these results show that soluble variants of both Aβ(1–40) and Aβ(1–42) internalise into SH-SY5Y cells via temperature and energy-dependent pathways, consistent with endocytosis.

### Dynamin-dependent effects on the uptake of Aβ(1–40) and Aβ(1–42)

**Figure 3:**
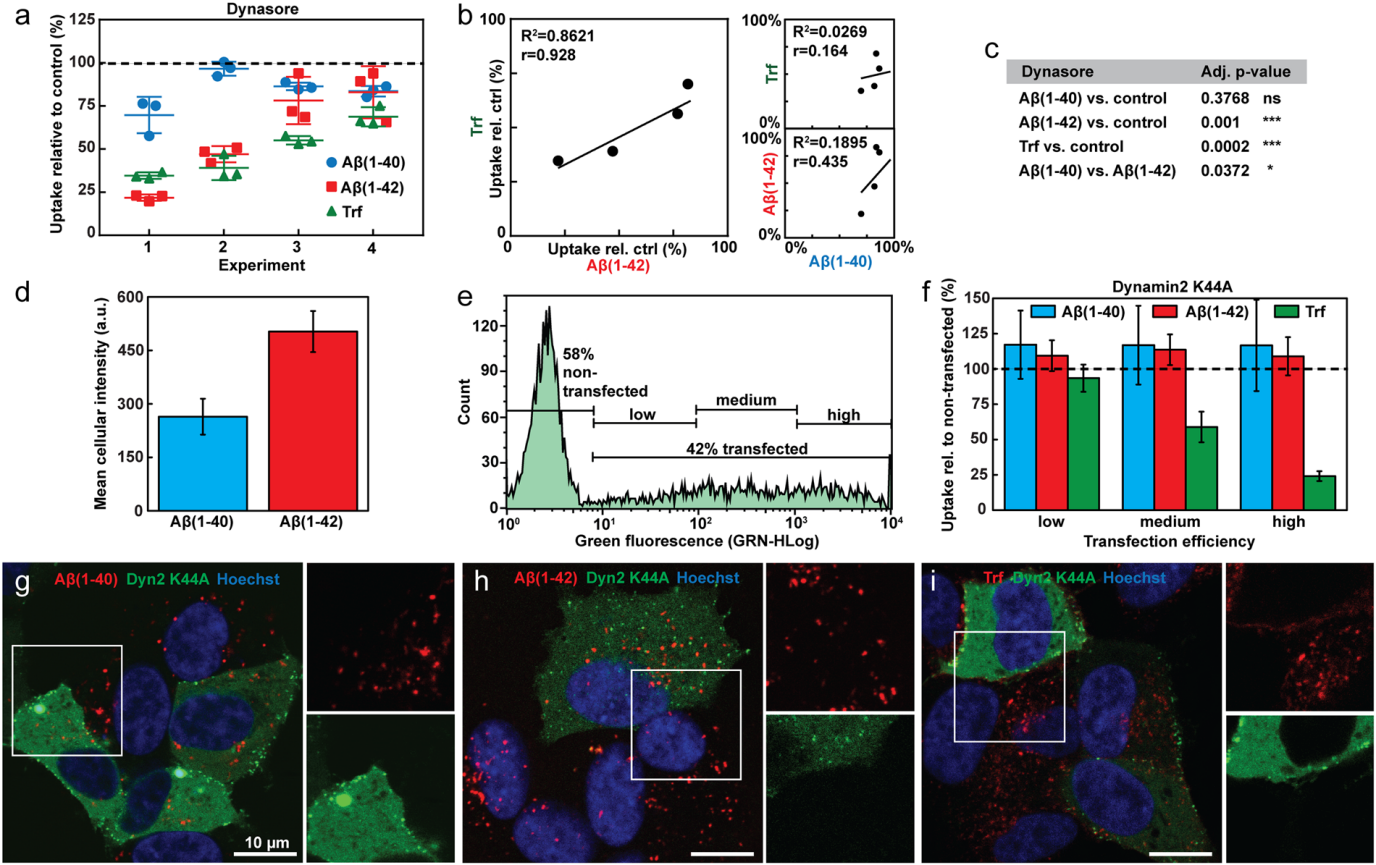
Uptake of Aβ(1–40), Aβ(1–42) and Trf in SH-SY5Y cells under conditions that perturb dynamin dependent endocytosis. (**a**) Uptake of HF488-labelled Aβ(1–40), Aβ(1–42) and AF647-labelled Trf in cells treated with dynasore (80 µM). The peptide uptake is reported as mean cellular uptake relative to control (cells not treated with inhibitor) for 4 independent experiments, each performed in triplicate. (**b**) Correlation analysis of the data presented in (**a**) with R^2^ of the linear fit of average inhibitions levels from each experiment and treatment, and Pearson’s correlation coefficient (r). (**c**) Statistical analysis of the data in (**a**) performed by one-way ANOVA with matched data followed by multiple comparisons with Bonferroni post-hoc test. The table shows the adjusted p-values for the individual comparisons made (*p ≤ 0.05, **p ≤ 0.01, ***p ≤ 0.001). (**d**) Mean cellular intensity ± SD (N = 2, n = 3–5) of live cells after incubation with HF647-labelled Aβ(1–40) or Aβ(1–42). (**e**) Flow cytometry histogram of live cells 24 h post transfection with EGFP-labelled dyn2 K44A: the cells were gated for peptide uptake based on transfection efficiency and the extent of dyn K44A overexpression as measured by the intensity of the EGFP label. (**f**) Uptake of HF647-labelled Aβ(1–40), Aβ(1–42) or AF647-labelled Trf in dyn2 K44A transfected cells, gated as in (**e**) and related to peptide uptake in cells gated as non-transfected (N = 2, n = 3–5). (**g**–**i**) Confocal imaging of dyn2 K44A (green) transfected cells imaged 24 h post transfection and incubated with HF647-labelled (**g**) Aβ(1–40), (**h**) Aβ(1–42) or (**i**) AF647-labelled Trf (red). In all experiments the concentration of Aβ was 1 μM and the concentration of Trf was 5 µg/ml. Cells were incubated with Aβ for 1 h and Trf for 5 min (flow cytometry) or 10 min (confocal microscopy). Relative uptake was calculated based on mean cellular fluorescence intensity ± SD of the total number of gated live cells measured by flow cytometry. All flow cytometry data were corrected for baseline contributions by subtracting the cellular autofluorescence.

We next explored how different endocytic mechanisms contribute to Aβ(1–40) and Aβ(1–42) uptake by subjecting SH-SY5Y cells to different pharmacological and genetic inhibitors of endocytosis, first focusing on dynamin-dependent uptake paths. Transferrin (Trf), a well-known target of clathrin-mediated endocytosis^34^, was included as reference and to probe the specificity and efficiency of the different treatments. We first used dynasore to perturb dynamin and hence vesicle scission^35^. In Fig. 3a we report the results of four independent experiments performed on different batches of cells and with several months in between the first and last experiment. We analysed the data using an one-way ANOVA with matched data, finding statistically significant variances between the sample means (p < 0.0001). This analysis was followed up by post-hoc tests with Bonferroni correction for the number of multiple comparisons given in Fig. 3c. Taken together our data and analysis show that Aβ(1–42), but not Aβ(1–40) uptake is statistically significantly reduced by dynasore inhibition in SH-SY5Y cells. There is also a small but significant (p = 0.0372) difference between Aβ(1–40) and Aβ(1–42) (Fig. 3c); a similar observation has been reported by Omtri *et al*.^36^ in PC-12 cells. We observed a correlative trend between Aβ(1–42) and Trf uptake inhibition, but not between Aβ(1–40) and Trf or indeed between Aβ(1–40) and Aβ(1–42) (Fig. 3b; the correlation was evaluated by computing the Pearson’s correlation coefficient). Despite extensive optimisation, dynasore treatment resulted in highly variable levels of Trf uptake inhibition in SH-SY5Y cells (Fig. 3a, 30–65%). This is likely a consequence of dynasore’s inhibition dependency on cell confluence, with decreasing efficacy at higher cell densities^35^. Since dynasore-induced inhibition of Trf uptake was significant, relative to untreated control in each independent experiment (two sample t-test, p-values 1.92e^−5^; 2.34e^−4^; 0.00116; 0.01514), we found no grounds for excluding experiments with low Trf inhibition, even though these also lead to a less efficient inhibition of Aβ(1–42).

To further examine dynamin-dependent effects on Aβ(1–40) and Aβ(1–42) uptake we transfected SH-SY5Y cells with an EGFP-labelled dominant negative mutant of dynamin2, K44A (dyn2 K44A) with impaired GTPase activity^37^. In order to quantify Aβ uptake simultaneously with dyn2 K44A overexpression (monitored by EGFP fluorescence) we switched to Hilyte Fluor™ 647 (HF647)-labelled Aβ peptides. Their relative uptake efficiency in non-transfected cells is comparable to that of the HF488-labelled peptides (Fig. 3d). The dyn2 K44A transfection efficiency was ~40% as shown from the flow cytometry histogram, moreover, the degree of overexpression in the transfected cells varied greatly (Fig. 3e). This is also evident in the confocal images shown in Fig. 3g–i. The EGFP-positive cells were therefore gated as low, medium or high expressing for further analysis. Next, the transfected cells were analysed for Aβ(1–40), Aβ(1–42) or Trf uptake and the signal was related to peptide uptake in cells in the non-transfected gate. The results show that whilst the uptake of Trf is inhibited in proportion to the degree of dyn2 K44A expression, no change in the uptake of Aβ(1–40) and Aβ(1–42) is observed (Fig. 3f). This observation was further verified by confocal microscopy; the images in Fig. 3g–i show that Aβ(1–40) (Fig. 3g) and Aβ(1–42) (Fig. 3h) display similar staining patterns in both transfected and non-transfected cells whereas intracellular transferrin is clearly less pronounced in the dyn2 K44A overexpressing cells (Fig. 3i).

### Cellular uptake of Aβ(1–40) and Aβ(1–42) under conditions that perturb clathrin-mediated endocytosis

**Figure 4:**
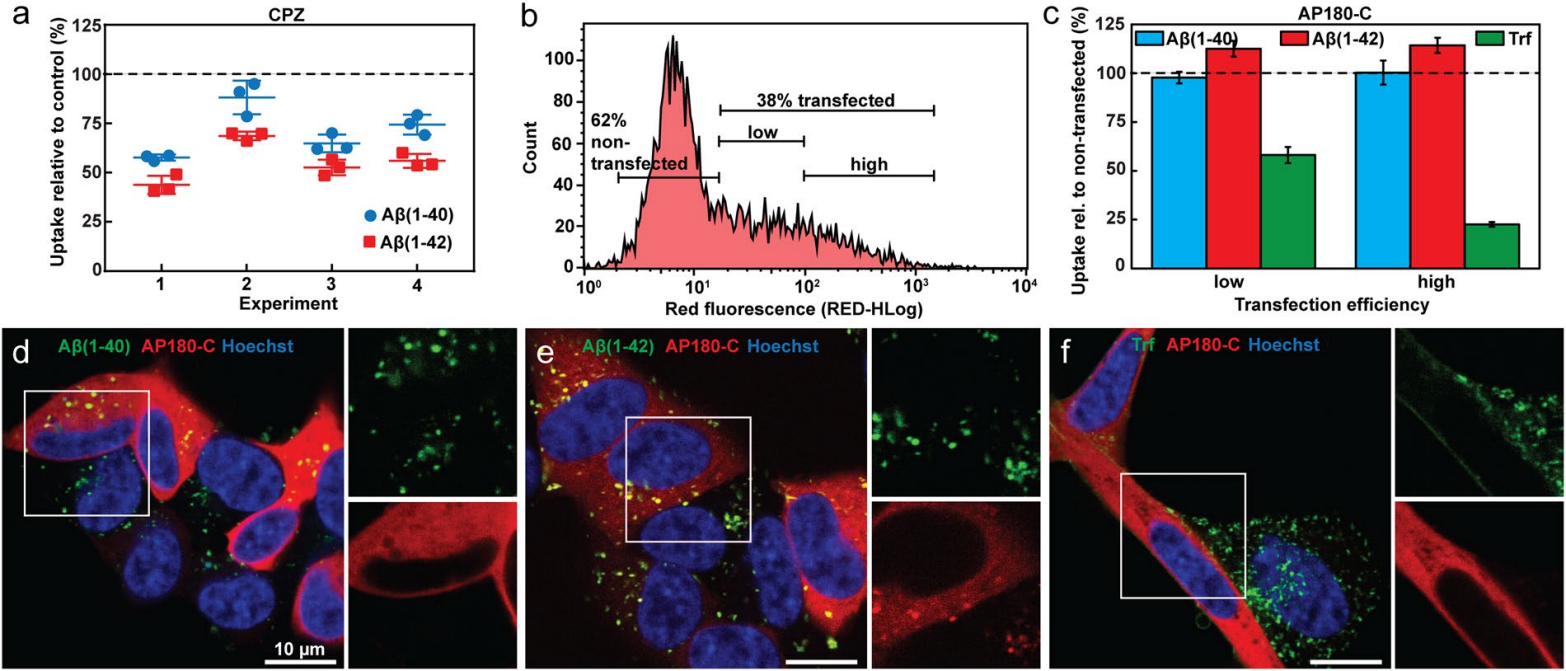
Uptake of Aβ(1–40), Aβ(1–42) and Trf in SH-SY5Y cells under conditions that perturb clathrin mediated endocytosis. (**a**) Uptake of HF488-labelled Aβ(1–40) and Aβ(1–42) in cells treated with CPZ (5 µg/ml). The peptide uptake is reported as mean cellular uptake relative to control (cells not treated with inhibitor) for 4 independent experiments, each performed in triplicate. Statistical analysis of the data performed by one-way ANOVA with matched data followed by multiple comparisons with Bonferroni post-hoc test gives adjusted p-values for the individual comparisons made as: Aβ(1–40) vs. control 0.0007; Aβ(1–42) vs. control <0.0001; Aβ(1–40) vs. Aβ(1–42) 0.0289. (**b**) Flow cytometry histogram of live cells 24 h post transfection with mRFP-labelled AP180-C: the cells were gated for peptide uptake based on transfection efficiency as measured by the intensity of the mRFP label. (**c**) Uptake of HF488-labelled Aβ(1–40), Aβ(1–42) or AF647-labelled Trf in AP180-C transfected cells, gated as in (**b**) and related to peptide uptake in cells gated as non-transfected (N = 2, n = 2–4). (**d**–**f**) Confocal imaging of AP180-C (red) transfected cells imaged 24 h post transfection and incubated with HF488-labelled (**d**) Aβ(1–40), (**e**) Aβ(1–42) or (**f**) AF488-labelled Trf (green). In all experiments the concentration of Aβ was 1 μM and the concentration of Trf was 5 µg/ml. Cells were incubated with Aβ for 1 h and Trf for 5 min (flow cytometry) or 10 min (confocal microscopy).

We also examined the uptake of Aβ upon perturbation of clathrin-mediated endocytosis. First we treated the SH-SY5Y cells with chlorpromazine (CPZ), an inhibitor of clathrin-coated pit assembly^38^. Under conditions where Trf uptake was inhibited by ~30% (see Supplementary Fig. S7) we also found reductions in Aβ(1–40) and Aβ(1–42) (Fig. 4a). The difference between Aβ(1–40) and Aβ(1–42) in mean uptake reduction was statistically significant in each separate experiment (two sample t-test, p-values 0.00796; 0.01839; 0.02407; 0.00675). The absolute levels of uptake reduction, however, varied between independent sets of experiments. We attribute this effect to the preparation and handling of CPZ; the solid powder is difficult to weigh accurately and CPZ solutions needs to be freshly prepared just prior to each experiment due to its rapid degradation in solution. In order to compare all the experiments, we performed an one-way ANOVA with matched data. The difference of means is significant (p < 0.0001) and was therefore followed by post-hoc tests with Bonferroni multiple comparison correction for the pairs specified in the figure text. Thus, Aβ(1–42) uptake is impaired to a greater extent than that of Aβ(1–40) following CPZ treatment.

To perturb clathrin-mediated endocytosis we next overexpressed an mRFP-labelled variant of the C-terminal of the clathrin-binding domain of AP180 (AP180-C)^39^. The transfection efficiency was ~40% (Fig. 4b). The extent of AP180-C overexpression in each cell varied, but not to the same extent as in the dyn2 K44A experiment (Fig. 3e); we therefore divided the mRFP positive cells into two gates (low and high) for further analysis (Fig. 4b). The uptake of Aβ(1–40), Aβ(1–42) or Trf in AP180-C transfected cells was thereafter quantified by flow cytometry and compared to the amount of peptide uptake in non-transfected cells (Fig. 4c). Whilst uptake of Trf systematically decreased with the degree of AP180-C overexpression, neither Aβ(1–40) nor Aβ(1–42) uptake was affected. In fact, overexpression of AP180-C resulted in a small but statistically significant increase in the uptake of Aβ(1–42) (two sample t-test, p-value 0.00113 and 0.00040 for low and high expression level, respectively). This could indicate that perturbation of clathrin mediated endocytosis leads to compensatory upregulation of Aβ(1–42) uptake paths. The results are consistent with the confocal microscopy images shown in Fig. 4d–f where transfected and non-transfected cells still display similar intracellular vesicular accumulation of Aβ(1–40) (Fig. 4d) and Aβ(1–42) (Fig. 4e), whereas for Trf the intracellular fluorescence is weaker in the transfected cells and to a higher extent localised to the plasma membrane (Fig. 4f).

### Aβ(1–40) and Aβ(1–42) uptake is reduced by macropinocytosis inhibition and does not involve Arf6-mediated endocytosis

**Figure 5:**
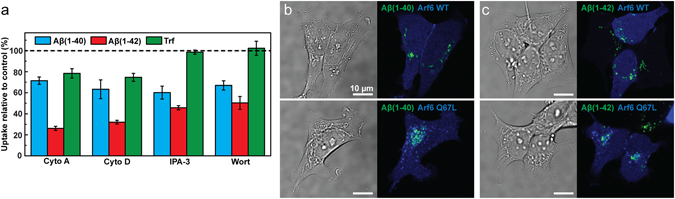
Uptake of Aβ(1–40), Aβ(1–42) and Trf in SH-SY5Y cells under conditions that perturb actin dependent endocytosis, macropinocytosis and Arf6-dependent endocytosis. (**a**) Uptake of HF488-labelled Aβ(1–40), Aβ(1–42) and AF647-labelled Trf in cells treated with 10 µM cytochalasin A (cyto A) and 25 µg/ml cytochalasin D (cyto D) to perturb actin polymerisation, or 10 µM IPA-3 and 25 nM wortmannin (wort) to perturb macropinocytosis. The peptide uptake is reported as mean cellular uptake relative to control (cells not treated with inhibitor) calculated based on mean cellular fluorescence intensity ± SD of the total number of gated live cells for three replicate samples (n = 3) measured by flow cytometry and corrected for baseline contributions by subtracting the cellular autofluorescence. (**b**,**c**) Images showing uptake of HF488-labelled Aβ(1–40) (**b**) and Aβ(1–42) (**c**) (green) in cells transfected with BFP-labelled Arf6 WT or Q67L (blue) alongside transmitted images showing the cells. The cells were incubated with Aβ 24 h post transfection. The Aβ concentration was 1 μM and the concentration of Trf was 5 µg/ml in all experiments. Cells were incubated with Aβ for 1 h (flow cytometry) or 24 h (confocal microscopy), and Trf for 5 min.

We next examined clathrin- and dynamin-independent cell uptake paths, first by exposure to cytochalasin A or D. These fungal metabolites destabilize actin filaments by inhibiting the addition of monomers to the filament ends^40^ and thereby perturb endocytic uptake, particularly via macropinocytosis^41^ where membrane ruffling is important to the formation of pinocytic vesicles^42^, but also via clathrin-mediated endocytosis^43^ although the mechanisms in the latter case are less clear. It was not possible to completely inhibit endocytosis with either cytochalasin A or D without severely compromising cell viability. However, under exposure conditions where >85% of the cytochalasin-treated cells were found in the live gate (see Supplementary Fig. S5) we observed ~30% reduction in the uptake of Aβ(1–40) and ~70% reduction in the uptake of Aβ(1–42) (Fig. 5a). Cytochalasin treatment also reduced the uptake of Trf, consistent with the involvement of actin in clathrin-mediated endocytosis. However, since we have already shown that clathrin-mediated endocytosis is not an important uptake path for either of the Aβ variants (Fig. 4c) it is likely that the reductions we observe with cytochalasin A and D are due to down-regulation of uptake via other endocytic paths.

We next treated cells with IPA-3^44^ or wortmannin^45^ that perturb macropinocytosis. These inhibitors also affected cell viability, but we could find exposure conditions that perturbed endocytosis and where at least 90% of the counted cells were gated as live (see Supplementary Fig. S5). EIPA, which is an alternative and common inhibitor of macropinocytosis was also tested, but we found it unspecific in SH-SY5Y cells regardless of the concentration used (25–200 µM). Treatment with IPA-3 and wortmannin reduced Aβ(1–40) uptake by ~50% and Aβ(1–42) uptake by ~60% under conditions where Trf uptake was identical to that in the control (Fig. 5a). This result therefore suggests that macropinocytosis contributes to the uptake of soluble forms of Aβ in neuron-type cells, thereby extending previous observations of the importance of this endocytic mechanism for the uptake of neurodegeneration-associated proteins in aggregated forms^46, 47^ and the observations of Aβ uptake in microglia^48^.

Finally, we tested if Aβ uptake is mediated via ADP ribosylation factor protein (Arf6) dependent endocytosis, which is a clathrin-independent and presumably dynamin-independent form of endocytosis which has been implicated in the cellular uptake of β-secretase and thus involved in the regulation of APP processing^49^. SH-SY5Y cells were transfected with wild-type Arf6 as well as plasmids carrying the GTP hydrolysis-resistant Q67L mutation, unable to recycle membrane back to the plasma membrane, thereby trapping Arf6 cargo in vacuolar structures^50^. Both variants were fused to BFP. We observed no difference in Aβ uptake between the wild-type and Q67L overexpressing cells (Fig. 5b,c), nor was there any apparent difference relative to the uptake in non-transfected cells. This suggest that macropinocytosis but not Arf6-dependent endocytosis is involved in Aβ(1–40) and Aβ(1–42) uptake.

## Discussion

Re-uptake of Aβ that has been secreted from neurons into the extracellular space can, alongside Aβ production and accumulation by the recipient cells themselves^22^, contribute to the intraneuronal build-up of Aβ peptides that has been observed to precede extracellular plaque formation in the Alzheimer disease afflicted brain^13–15^. We demonstrate that Aβ(1–40) and Aβ(1–42) are taken up into human neuroblastoma SH-SY5Y cells via constitutive and virtually non-saturable endocytic mechanisms that result in approximately twice as efficient internalisation of Aβ(1–42) compared to Aβ(1–40). Furthermore, we show that the endocytic uptake is largely independent of clathrin- and dynamin, but significantly reduced by inhibitors of actin polymerisation and macropinocytosis.

This study focused mainly on the differences in uptake efficiency between Aβ(1–40) and Aβ(1–42). In this context our observations expand previous work by Burdick *et al*.^27^, and also provides a quantitative estimate on intracellular Aβ concentrations; incubation of SH-SY5Y cells for 8 h with 1 μM solutions of monomeric (~85% as estimated by SDS-PAGE) peptide result in an intracellular accumulation of ~400,000 Aβ(1–40) molecules or ~800,000 Aβ(1–42) molecules. This corresponds to vesicular concentrations of respectively ~60 µM and ~140 µM which is at least an order of magnitude above the critical concentration for their self-assembly into amyloid fibrils. It is therefore not surprising that we and others have reported that Aβ uptake contribute to the accumulation of fibrils or other insoluble aggregates in endolysosomal compartments^23, 25, 51^. Our data suggest that Aβ(1–40) and Aβ(1–42) uptake differences arise due to differences in the ability of their soluble monomers to interact with uptake-promoting components of the cell surface. Hence, the two additional C-terminal hydrophobic residues in the primary sequence of the latter variant appear decisive for its superior uptake. We base this conclusion on that we observe differences in uptake already after 1 h; this time span is insufficient to lead to aggregation at the extracellular concentrations we supply (≤1 μM), even for Aβ(1–42)^52^ which has an intrinsic higher amyloid formation propensity than Aβ(1–40)^53^. Moreover, we do not observe extensive accumulation of the Aβ variants at the cell surface prior to uptake; accelerated amyloid growth or oligomer formation in this vicinity^54^ prior to uptake is thus unlikely. This is important since another study has shown that fibrillar forms of Aβ(1–42) are, in fact, taken up more efficiently than the corresponding soluble forms^48^.

Our study demonstrates conclusively that endocytosis is the major, if not only, pathway of entry for Aβ(1–40) and Aβ(1–42) into SH-SY5Y cells. This opposes some previous ideas of passive diffusion or membrane-penetration modes of entry, which would allow Aβ direct access to the cytoplasm^36, 55^. Our finding that Aβ(1–40) and Aβ(1–42) uptake is proportional to cellular endocytic rate is also in line with work by Friedrich *et al*.^51^ who demonstrated that the extent of Aβ(1–40) plaque formation in cell culture is proportional to the endocytic activity of the cells; thus there appear to be a robust connection between Aβ endocytosis and its aggregate formation in Alzheimer’s disease.

In the second part of this study we explored how different types of endocytosis contribute to the uptake of Aβ(1–40) and Aβ(1–42). The main conclusion in this respect is that both peptides largely use the same uptake paths. Our finding that their internalisation is both dynamin- and clathrin-indepedent is important given the high number of receptor proteins that have been suggested to mediate Aβ uptake and that utilise clathrin-mediated endocytosis to enter cells^56^. It is also surprising in relation to our finding that the uptake of Aβ(1–42) but not Aβ(1–40) is reduced in cells treated with the dynamin inhibitor dynasore; this result was fully in line with observations by Omtri *et al*. in PC-12 cells^36^ and consistent with our experiments with CPZ. A possible explanation to this discrepancy is if dynasore reduces Aβ(1–42) but not Aβ(1–40) by a dynamin-unrelated effect, e.g. via non-specific endocytosis inhibition. It has for example been proposed that dynasore can disturb plasma membrane cholesterol levels and thereby disrupt lipid rafts^57^. These membrane microdomains have been implicated in Aβ endocytosis^56^ and Aβ peptides are indeed enriched in brain lipid raft fractions obtained from Tg2576 AD mice^58^. We attempted to investigate this further by methyl-β-cyclodextrin induced cholesterol depletion. This was, however, not successful due to high levels of cytotoxicity. Another interesting observation is that Aβ(1–42), but not Aβ(1–40) uptake was increased in AP180-C overexpressing cells, suggesting that compensatory endocytic uptake mechanisms may come into play and that these are somehow selective towards Aβ(1–42). Such compensation has been suggested as a mechanism to retain cell surface area^59^, Damke *et al*. have observed upregulation of fluid-phase endocytosis in response to dynamin-1 inhibition^60^ and we have previously observed that stimulation of macropinocytosis in CHO-K1 cells did not increase membrane turnover^61^.

We demonstrate that both Aβ(1–40) and Aβ(1–42) use actin-dependent endocytic mechanisms to enter cells. The uptake is consistent with macropinocytosis based on the Aβ-specific (as opposed to Trf) inhibitory effects of IPA-3 and wortmannin. Macropinocytosis has been reported to be important in the clearance of Aβ(1–42) by microglia^48^ and has also been implicated in the prion-like transmission of aggregated forms of other amyloidogenic proteins^46, 47^. The involvement of this path is intriguing because of suggestions that macropinosomes experience slow transport rates^62^, may skip fusion with early endosomes^63^ and may be particularly leaky which could contribute to augment protein aggregate toxicity^64^.

Taken together, our study suggests that Aβ(1–40) and Aβ(1–42) are internalised into cells via predominately clathrin- and dynamin-independent endocytosis and via uptake paths that are consistent with macropinocytosis but not with Arf6-mediated endocytosis. We also note interesting differences in uptake between the two Aβ variants following presumably off-target effects of dynasore and CPZ as well as an up-regulation of Aβ(1–42) uptake in AP180-C overexpressing cells. Albeit the biological reasons for this differences remain unexplained our observations indicate that additional dynamin-independent uptake paths may exist for Aβ(1–42). Our results are important for how Aβ re-uptake may contribute to intraneuronal Aβ accumulation and thus contribute to our cellular and molecular understanding of early pathological traits in the development of Alzheimer’s disease.

## Methods

### Reagents

Synthetic Aβ(1–40) and Aβ(1–42) peptides, conjugated to the HiLyte Fluor™ (HF) fluorophore HF488 or HF647 at the N-terminus, were from Anaspec Inc. (Fremont, US). Unless else is noted experiments were performed with HF488-labelled peptides. The peptide purity was >95% as determined by Anaspec Inc. by MS and RP-HPLC. AlexaFluor488 (AF488)- and AlexaFluor647 (AF647)-labelled Transferrin (Trf), AF488-labelled dextran 10 kDa, FM 4–64, Hoechst 33342, LysoTracker Deep Red, calcein-AM and propidium iodide were from Molecular Probes and purchased via ThermoFisher Scientific (Gothenburg, Sweden). NuPAGE Novex 4–12% Bis-Tris protein gel and 1% MES running buffer were from Invitrogen and purchased via ThermoFisher Scientific. The endocytosis inhibitors cytochalasin A, cytochalasin D, dynasore, wortmannin, 1,1′-Disulfanediyldinaphthalen-2-ol (IPA-3), and chlorpromazine hydrochloride (CPZ) were from Sigma Aldrich (Stockholm, Sweden). The human neuroblastoma cell line SH-SY5Y was purchased from Sigma Aldrich, the Chinese hamster ovary cell line CHO-K1 was a kind gift from Prof. S. Lange, Gothenburg University and the murine fibroblast cell line NIH 3T3 was acquired from ATCC (Wezel, Germany). Cell culture reagents (minimal essential medium, nutrient mixture F-12 Ham, MEM non-essential amino acids, Dulbecco’s High Glucose Modified Eagles Medium, heat-inactivated fetal bovine serum, calf bovine serum, L-glutamine, trypsin-EDTA 0.05% and Accutase) and buffers (HEPES and PBS) were from PAA Laboratories GmbH (Pashing, Austria) or HyClone (GE Healthcare, South Logan, US), and B-27 was from Gibco/Life Technologies and purchased via ThermoFisher Scientific. Plasmids encoding for dynamin2-K44A-EGFP (dyn2 K44A), mRFP- AP180-C (C-terminal residues 530–915 of AP180; AP180-C), and Arf6-WT-BFP and Arf6-Q67L-BFP were kindly provided by Dr. Frode Miltzow Skjeldal (dyn2 K44A; University of Oslo), Assoc. Prof. Björn Granseth (AP180-C; Linköping University) and Assoc. Prof. Richard Lundmark (Arf6 WT and Q67L; Umeå University).

### Preparation and handling of Aβ peptides

The lyophilized Aβ peptide powders were dissolved in hexafluoro-2-propanol to disrupt any aggregates^26^ and monomerize the peptide. The solutions were vortexed briefly and aliquoted at 4 °C. The solvent in each aliquot was evaporated at 37 °C for 60 min using a RVC 2–18 CD Rotational Vacuum concentrator (Martin Christ, Germany). The remaining peptide films were snap frozen in liquid nitrogen and kept at −80 °C until further use. For concentration determinations the peptide film was dissolved in 1% ammonium hydroxide (v/v) and the absorption of the dye label was measured on a Cary 4000 UV-Vis Spectrophotometer (Agilent Technologies, Santa Clara, CA, US). Extinction coefficients of 70,000 M^−1^cm^−1^ at 504 nm and 250 000 M^−1^cm^−1^ at 649 nm was used for the HF488 and HF647 dye labels, respectively, according to the information provided by the manufacturer. Prior to each experiment one peptide film was dissolved in a small volume 1% ammonium hydroxide (v/v) and diluted with cell culture medium supplemented with 30 mM HEPES. The concentration of ammonium hydroxide was kept below 0.03% and was matched in controls to ensure identical treatment of all samples. Unused samples were discarded in order to avoid Aβ aggregation induced by freeze-thawing. To confirm that the peptide solutions supplied to cells contained soluble, mainly monomeric, material we prepared a 1 µM peptide solution in cell culture media as described above, followed by separation on a NuPAGE Novex 4–12% Bis-Tris protein gel in 1% MES running buffer at 200 V 130 mA for 35 min. The gel was scanned in a Typhoon 9410 Variable Mode Imager gel scanner (GE Healthcare, Princeton, NJ, US). The HF488 dye was excited by a 488 nm laser and emission detected through a 520/40 nm filter. After background correction, the gel bands corresponding to monomer, dimer and trimer species were quantified in ImageJ (NIH, Bethesda, Maryland, US) by integrated intensity of the respective bands.

### Cell culture and sample preparation

SH-SY5Y cells were grown in a 1:1 mixture of minimal essential medium (MEM) and nutrient mixture F-12 Ham supplemented with 10% heat-inactivated fetal bovine serum, 1% MEM non-essential amino acids and 2 mM L-glutamine. The cells were detached (trypsin-EDTA 0.05%, 5 minutes) and passaged twice a week. Cells were plated 1day prior to experiments in either flat-bottomed 96 well plates (Nunc; 60,000 cells/well for experiments <1day and 20,000 cells/well for experiments ≥1day) for flow cytometry or in glass-bottomed culture dishes (MatTek; 25,000 cells/14 mm dish) for microscopy.

CHO-K1 cells were grown in nutrient mixture F-12 Ham supplemented with 10% heat-inactivated fetal bovine serum and 2 mM L-glutamine. The cells were detached (trypsin-EDTA 0.05%, 7 minutes) and passaged twice a week. NIH 3T3 cells were grown in Dulbecco’s High Glucose Modified Eagles Medium (DMEM) with 4 mM L-glutamine, 4500 mg/ml glucose and sodium pyruvate, supplemented with 10% calf bovine serum. The cells were detached (cell detachment solution Accutase, 1–2 minutes) and passaged twice a week. When included in the same experiment, SH-SY5Y, CHO-K1 and NIH 3T3 cells were plated in flat-bottomed 96 well plates (Nunc; 40,000 cells/well 24 h prior to experiment) for flow cytometry or in glass-bottomed culture dishes (MatTek; 20,000 cells/14 mm dish 48 h prior to experiment) for microscopy.

### Confocal microscopy and image analysis

The samples were washed 1x in serum free medium prior to incubation with Aβ peptides and/or other reagents also in serum free medium that was supplemented with 30 mM HEPES and 2% B-27. For experiments including Trf B-27 was omitted as it, according to the media formulation provided by the manufacturer, contains unlabelled Trf that interferes with the experiment. Prior to imaging, cells were stained with 50 nM LysoTracker Deep Red for 30 min and 5 µg/ml Hoechst 33342 was added during the last 10 min of incubation. The cells were thereafter washed 1x, treated with 5 µg/ml FM 4–64 and imaged immediately (for labelling of cellular membrane) or after 10 min incubation at 37 °C (for labelling of cellular membrane and endocytic vesicles). Confocal images were acquired either on a Nikon C2+ confocal microscope equipped with a C2-DUVB GaAsP Detector Unit and using an oil-immersion 60 × 1.4 Nikon APO objective (Nikon Instruments, Amsterdam, Netherlands), or on a home-built Thorlabs CLS system equipped with a Galvo:Resonant scanner, a Multi-Channel Fiber-Coupled laser source and High-Sensitivity GaAsP PMT detectors recording onto ThorImageLS software (Thorlabs Inc, New Jersey, U.S.A.). The scanner unit was mounted onto a Leica DMIRB microscope equipped with an oil immersion 63x NA 1.47 Leica HCX PL APO objective for imaging and co-localisation analysis, or a 40x NA 0.55 Leica N PLAN L CORR air objective for quantification of peptide uptake. The sample was excited and detected sequentially with appropriate excitation laser lines and emission filters.

For quantification of peptide uptake by confocal microscopy, cells were incubated with 1 µM Aβ(1–40) or Aβ(1–42) as described above, washed to remove extracellular peptide and stained with 5 µg/ml FM 4–64 and 5 µg/ml Hoechst. Images were captured with an open pinhole, corresponding to 7.8 airy units (AUs), in order to sample intensity from intracellular peptide throughout the full thickness of the cells. First, to determine an accurate sampling depth, images were acquired at three focal planes separated by 1 µm between each and analysed for matched summed intracellular intensity. Next, images were acquired with identical settings from a field of view with on average 25 cells/image. Image analysis was performed in the ImageJ software (NIH, Bethesda, Maryland, US). To mask out the regions of interest, Gaussian blur was applied to the Aβ channel, followed by background subtraction using the in-built function in ImageJ. The resulting image was thresholded at 5%, and converted to a binary image with background set to 0 and regions of interest (corresponding to Aβ containing intracellular regions) set to 1. The binary mask image was compared to the FM 4–64 channel to confirm that all masked regions correspond to intracellular locations. The original Aβ channel was thereafter treated by background subtraction and multiplication by the binary mask. To obtain the total Aβ emission intensity per cell, all pixel values were summed and divided by the total number of cells in the image (the cell number was determined from the number of nuclei visible in the Hoechst emission channel). In order to convert total cellular intensity to number of emitting molecules, a calibration curve was established by imaging of Aβ solutions diluted to known concentrations (0–10 µM). The solutions were sandwiched between two cover slips separated by 38 µm Scotch Magic Tape in order to obtain a defined sample depth. All images for the calibration curve were obtained with identical laser power, detector gain and pixel size as for cellular imaging, but with a smaller pinhole size corresponding to 1.0 AU. The mean intensity per pixel was calculated and related to concentration by performing a linear regression of mean intensity from three samples per concentration. The imaging depth was calculated based on imaging parameters and objective specifications generating a point spread function (PSF) for the focal spot. The resulting voxel size was calculated to be 740 nm in the axial direction, whereby 2.5x this voxel was determined to be representative of the sampling probe depth. This value combined with the linear regression was used to determine the intensity per Aβ molecule under the applied imaging conditions. Intensity per molecule was then used to convert the summed intensity per cell to the number of internalised Aβ molecules per cell. 16 images (8 images per cell culture dish, experiment performed in duplicate) with a total of ~450 cells were analysed for both Aβ(1–40) and Aβ(1–42), and the data is presented as mean ± SD.

For co-localisation analysis of the Aβ peptides with LysoTracker Deep Red, the cells were incubated with either Aβ(1–40) or Aβ(1–42) for a period of 24 h. The cells were thereafter washed and incubated for an additional 5 h, stained with 50 nM LysoTracker Deep Red and 5 µg/ml Hoechst and then imaged. Images were acquired with identical settings, with on average 5 cells in each field of view. The images were analysed in the ImageJ software (NIH, Bethesda, Maryland, US) with the plug-in JACoP^28^. Prior to co-localisation analysis, images were pre-treated in ImageJ by automatic background subtraction and removal of outliers. In the JACoP plugin, the Aβ and LysoTracker DeepRed channels were thereafter thresholded automatically followed by quantification of signal overlap by Mander’s coefficient (fraction of Aβ overlapping LysoTracker Deep Red). 21 images (7 images per cell culture dish, experiment performed in triplicate) were analysed for both Aβ(1–40) and Aβ(1–42), and the data is presented as mean ± SD.

### Flow cytometry

Flow cytometry was used to quantify cellular uptake. The samples were washed in serum free medium (1x for experiments including only Aβ and 3x for experiments with Trf) prior to incubation with Aβ peptides and/or other reagents in serum free medium supplemented with 30 mM HEPES and 2% B-27. For experiments including Trf B-27 was omitted, as outlined above. Prior to analysis, the cells were washed 1x (for experiments including only Aβ) or 3x (for experiments including Aβ and dextran 10 kDa or Trf) in serum free medium, detached by trypsin-EDTA for 10 min followed by addition of FBS-supplemented cell culture medium to inhibit further proteolytic degradation. All samples were kept on ice until they were analysed on a Guava EasyCyte 8HT (Millipore, Darmstadt, Germany) that automatically retrieves samples from a 96-well plate. In order to exclude effects due to difference in delay time we used mixed order of analysis, loading only a few samples at a time with the remaining samples kept on ice. The cell counting gate was determined using propidium iodide (0.5 µM, not washed away prior to analysis) and calcein-AM (10 nM, 15 min staining) to distinguish live and dead cells, and for each sample 5,000 cells from within the live gate were counted and analysed. HF488, AlexaFluor488, EGFP and calcein-AM were excited by a 488 nm laser and their fluorescence was detected through a 525/30 nm filter. Propidium iodide and mRFP were excited by the 488 nm laser but detected through a 692/46 nm filter. HF647 and AlexaFluor647 were excited with a 635 nm laser and detected through a 661/19 nm filter. Compensation was performed to adjust for bleed through in experiments including mRFP. Mean cellular uptake was estimated as the average fluorescence intensity of all cells gated within the live region and all individual experiments were performed with three technical replicates (n = 3) unless else is stated. The average fluorescence intensity was baseline corrected by subtracting the signal for unstained cells. All flow cytometry data was analysed in InCyte software (Millipore, Darmstadt, Germany) and displayed using FlowJo software (Tree Star, Inc., San Carlos, CA, US).

### Perturbation of endocytosis in SH-SY5Y cells

#### Endocytosis perturbation by low temperature and ATP depletion

Endocytic uptake was inhibited or perturbed by several methods, including incubation at 4 °C and ATP depletion. Incubation at 4 °C was used to shut down all endocytic uptake paths^31^. Cells were washed 1x with ice-cold serum-free medium prior to incubation with 500 nM Aβ solutions above ice. The cells were thereafter washed 1x and immediately subjected to flow cytometry analysis, or transferred to 37 °C for a 2 h recovery period before analysis. Control cells were kept at 37 °C. Depletion of ATP was used to perturb active, energy-dependent, uptake paths. Cells were washed 1x with PBS solution supplemented with Ca^2+^ and Mg^2+^ and thereafter pre-incubated with an ATP depletion solution (10 mM 2-deoxy-D-glucose and 10 mM sodium azide in PBS) for 2 h. 1 µM Aβ, diluted in the ATP depletion solution, was added and the cells were incubated for 1 h prior to flow cytometry analysis. It was not possible to use longer incubation times as this was associated with intolerable levels of toxicity and cell death. We controlled for this by testing the cells’ abilities to recover from ATP depletion. In this case, cells were pre-incubated with ATP depletion solution for 3 h (total duration in the above experiment), washed 1x in serum-free medium and thereafter incubated with 1 µM Aβ diluted in serum-free medium for 2 h (recovery) prior to flow cytometry analysis. Cells cultivated in serum-free medium throughout the experiment were used as controls.

#### Pharmacological inhibition of endocytic mechanisms

Endocytic mechanisms were also perturbed by treatment of cells with pharmacological inhibitors. Stock solutions of cytochalasin A, cytochalasin D, dynasore, wortmannin and IPA-3 were prepared in DMSO and diluted in serum-free cell culture media supplemented with 30 mM HEPES on the day of the experiment. The final DMSO concentration added to the cells was kept <0.1% and matched for the control. Prior to addition of Aβ peptide the cells were always washed in serum-free medium followed by pre-incubation for 30 min with either of the inhibitors diluted in serum-free medium with the addition of 30 mM HEPES. 1 µM Aβ was thereafter added and the cells were incubated for a total of 1 h, in presence of the respective inhibitors. The cells were centrifuged 1000 * *g* for 5 min at 4 °C, washed 3x with ice-cold serum-free medium, followed by detachment and immediate flow cytometry analysis, as detailed above. We could not completely inhibit endocytosis with pharmacological inhibitors without severely compromising cell viability. However, under exposure conditions where at least 75% if the inhibitor-treated cells were found in the live gate (see Supplementary Fig. S5) we observed reductions in Aβ uptake. Only the live cells were included in the analysis. Trf was used as a positive control for clathrin-mediated endocytosis^34^. Due to the rapid uptake and recycling of internalized Trf ^65^ we adopted an optimized protocol with sufficiently short Trf incubation times. First, to remove the quantities of membrane-bound Trf that originate from the serum component of the complete culture medium the cells were washed 3x in serum-free medium. The cells were then subjected to the inhibitors as described above and thereafter exposed to a 5 min pulse of 5 µg/ml Trf. To remove externally bound fluorescently labelled Trf the cells were thereafter washed (3x/2 min) in ice-cold acidic buffer (0.1 M Glycine-HCl buffer pH 2.5 with 150 mM NaCl)^35^ whilst kept on ice. The cells were then harvested for flow cytometry analysis, and analysed one sample at a time with mixed order of analysis whilst keeping the remaining samples on ice. Since the short 5 min pulse treatment with Trf differs with respect to the total inhibitor exposure time compared to the 60 min incubation with Aβ, we tested if inhibitor exposure time would influence the extent of Trf cellular uptake by adding Trf for 5 minutes at different time points. None, or only minor effects that would not influence the use of Trf as a control for clathrin-mediated endocytosis, were observed (see Supplementary Fig. S6). Due to observed instability of CPZ in aqueous media it was always prepared fresh in cell culture medium immediately before each experiment. To ensure that CPZ was not degraded and thus actively blocking clathrin-mediated endocytosis throught the timespan of the experiment, Trf was added to the cells as a 5 min pulse at the end of the 1 h Aβ incubation, whereupon the protocol for Trf described above was followed. Once a suitable CPZ concentration had been established (see Supplementary Fig. S7), a larger set of experiments were performed with Aβ solely, applying the protocol for Aβ.

Prior to performing the experiments with Aβ we optimized extensively the inhibitor concentrations needed to perturb endocytosis without compromising cell viability. The following concentrations were found optimal for the SH-SY5Y cell line and the in total up to 90 min exposure time that was needed for our experiments: 80 µM dynasore, 5 µg/ml CPZ, 10 µM cytochalasin A, 25 µg/ml cytochalasin D, 25 nM wortmannin and 10 µM IPA-3.

#### Perturbation of endocytosis by transfection with AP180-C, dyn2 K44A and Arf6 WT/Q67L

SH-SY5Y cells were passaged two days prior to transfection, grown to ~70% confluency and transformed with plasmids encoding for AP180-C, dyn2 K44A or Arf6 WT/Q67L by electroporation using a Neon Transfection System (Invitrogen, Carlsbad, CA, US), following the protocol provided by the manufacturer and applying a single pulse of 1,100 V with a pulse width of 50 ms. The cells were transfected using 1–2.5 µg plasmid DNA/100,000 cells in a 10 µl Neon Tip and plated immediately after, 24 h prior to experiment, in flat-bottomed 96 well plates (Nunc; 50,000 cells/well) for flow cytometry or in glass-bottomed culture dishes (MatTek; 100,000 cells/14 mm dish) for microscopy. Prior to experiment, the cells were washed, exposed with Aβ(1–40), Aβ(1–42) or Trf and imaged by confocal microscopy or harvested for flow cytometry analysis as detailed under the above section *Pharmacological inhibition of endocytic mechanisms*.

### Statistics

All flow cytometry data are presented as mean ± standard deviation (SD) of at least three replicate samples (n = 3). Statistical analysis was performed by two-tailed two-sample t-tests (alpha level 0.05) using Origin software (OriginLab, Northampton, MA, US) or matched sample ANOVA using GraphPad Prism software (GraphPad, San Diego, CA, US). Matched sample ANOVA was followed by multiple comparisons with Bonferroni post-hoc test to test for differences in mean peptide uptake between specific samples. This means that the reported individual p-values have been adjusted for the number of comparisons that were relevant to the experiment (according to Bonferroni). We also applied the GraphPad Prism software to calculate Pearson’s correlation coefficient (r) in the analysis of peptide uptake in between independent experiments with the dynasore inhibitor.

## Electronic supplementary material

Supplementary Information
