# Supplementary material for: Endocytic uptake of monomeric amyloid-β peptides is clathrin- and dynamin-independent and results in selective accumulation of Aβ(1–42) compared to Aβ(1–40)
Source: Sci Rep. 2017 May 17;7:2021. doi: 10.1038/s41598-017-02227-9 (PMC5435687; doi:10.1038/s41598-017-02227-9)
Supplement: Supplementary file 1 — Supplementary Information [file 41598_2017_2227_MOESM1_ESM.pdf]

Supplementary Information

Endocytic uptake of monomeric amyloid-β peptides is clathrin- and dynamin-independent and results in selective accumulation of Aβ(1-42) compared to Aβ(1-40)

Emelie Wesén[1], Gavin D. M. Jeffries[2], Maria Matson Dzebo[1,2], Elin K. Esbjörner[1,*]

[1]Division of Chemical Biology, Department of Biology and Biological Engineering, Chalmers University of Technology, Kemivägen 10, 412 96 Gothenburg, Sweden

[2]Division of Chemistry and Biochemistry, Department of Chemistry and Chemical Engineering, Chalmers University of Technology, Kemivägen 10, 412 96 Gothenburg, Sweden

*Corresponding author; e-mail: eline@chalmers.se, phone: +46 (0)31 772 5120

1. Fluorescence emission intensity of HF488 labelled Aβ peptides

The emission intensity was measured on 1 μM Aβ solutions to confirm equal emission intensities of the two fluorophore-conjugated Aβ variants at the same peptide concentration (Fig. S1). Spectra were recorded in a 60 µl, 3 mm path length, quartz micro-cuvette (Hellma Analytics, Müllheim, Switzerland) using a Varian Eclipse fluorimeter (Agilent Technologies, Santa Clara, CA, US). The samples were excited at 470 nm and the emission was collected between 480-700 nm.

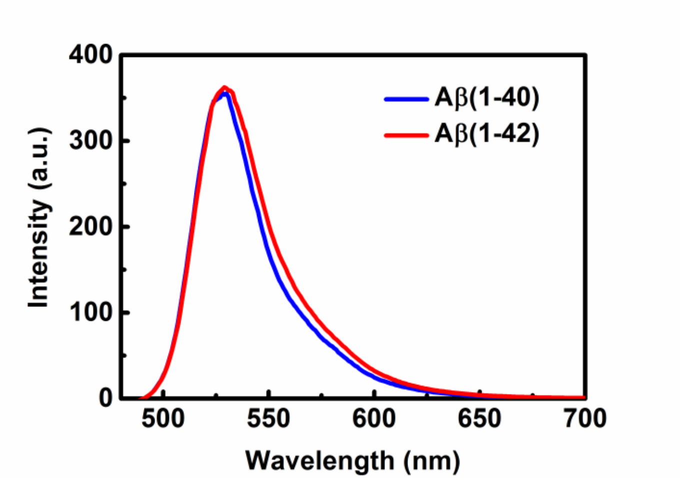

Supplementary Figure S1. *Emission spectra of HF488 labelled Aβ(1-40) and Aβ(1-42) at a peptide concentration of 1 µM. The data is presented as average fluorescence emission intensity of two aliquots each of Aβ(1-40) and Aβ(1-42).*

2. Calibration curve for quantification of amyloid-β in SH-SY5Y cells

Quantification of intracellular Aβ(1-40) and Aβ(1-42) was performed using confocal imaging, through establishment of a calibration curve using Aβ solutions with known concentrations ranging from 0-10 μM. The procedures are described in the Methods section of the main text. The calculated average pixel intensity as a function of peptide concentration is shown in Fig. S2. The images were recorded with a pixel size of 152 nm*152 nm and a probe depth of 1.85 μm (corresponding to 2.5 axial voxels). Using these numbers, the average pixel intensity was translated to a mean intensity per Aβ molecule of 5.67. This value was thereafter used to quantify cellular uptake as displayed in Fig. 1D and explained in the main text.

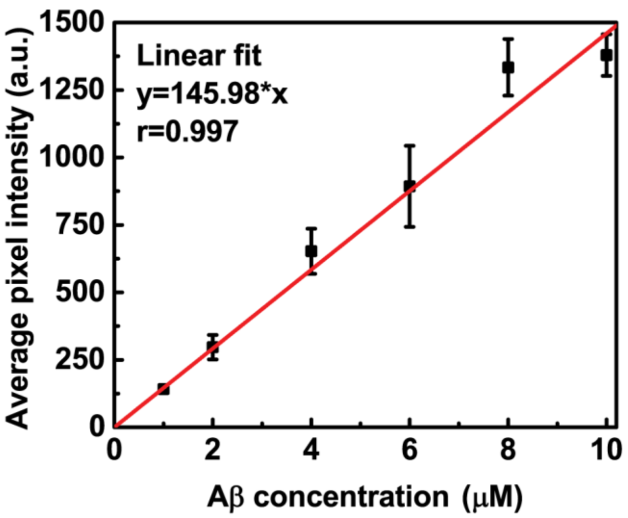

Supplementary Figure S2. *Calibration curve for quantitative confocal microscopy analysis. Aβ solutions with known concentrations were sandwiched between two coverslips separated by 38 μm Scotch Magic Tape and imaged as described in the Methods section. Data is presented as average fluorescence intensity per pixel (±SD) (n=3).*

3. Identification of the live cell cluster for flow cytometry analysis

In the quantitative peptide uptake experiments analysed by flow cytometry it was advantageous to only include viable cells in the analysis, as dead cells could have compromised membranes that may influence the extent of uptake, thereby biasing the results. It was also important to establish an experimental treatment window for endocytosis perturbation, whereby the inhibitors had significant effects without being highly cytotoxic. Supplementary Fig. S3A-B shows the localisation of live and dead cells on flow cytometry FSC/SSC dot-plots. The gate corresponding to live cells was set based on the cluster of untreated cells (Supplementary Fig. S3A), whereas the gate corresponding to dead cells was based on the main cluster following treatment with 0.1% H_2O_2 (Supplementary Fig. S3B). Cells were also stained with calcein-AM (live stain) and propidium iodide (dead stain). The corresponding intensity histograms confirmed the position of the live and dead gates (Supplementary Fig. S3C-D). Only cells within the live gate were analysed in the quantitative uptake experiments. Treatment with Aβ alone at the concentration and incubation times used in this study did not result in an apparent shift of the cell population in the FSC/SSC dot-plots. The cell cluster is still concerted and localised to the live cell gate after a 5 h incubation with 1 μM Aβ (Supplementary Fig. S4).

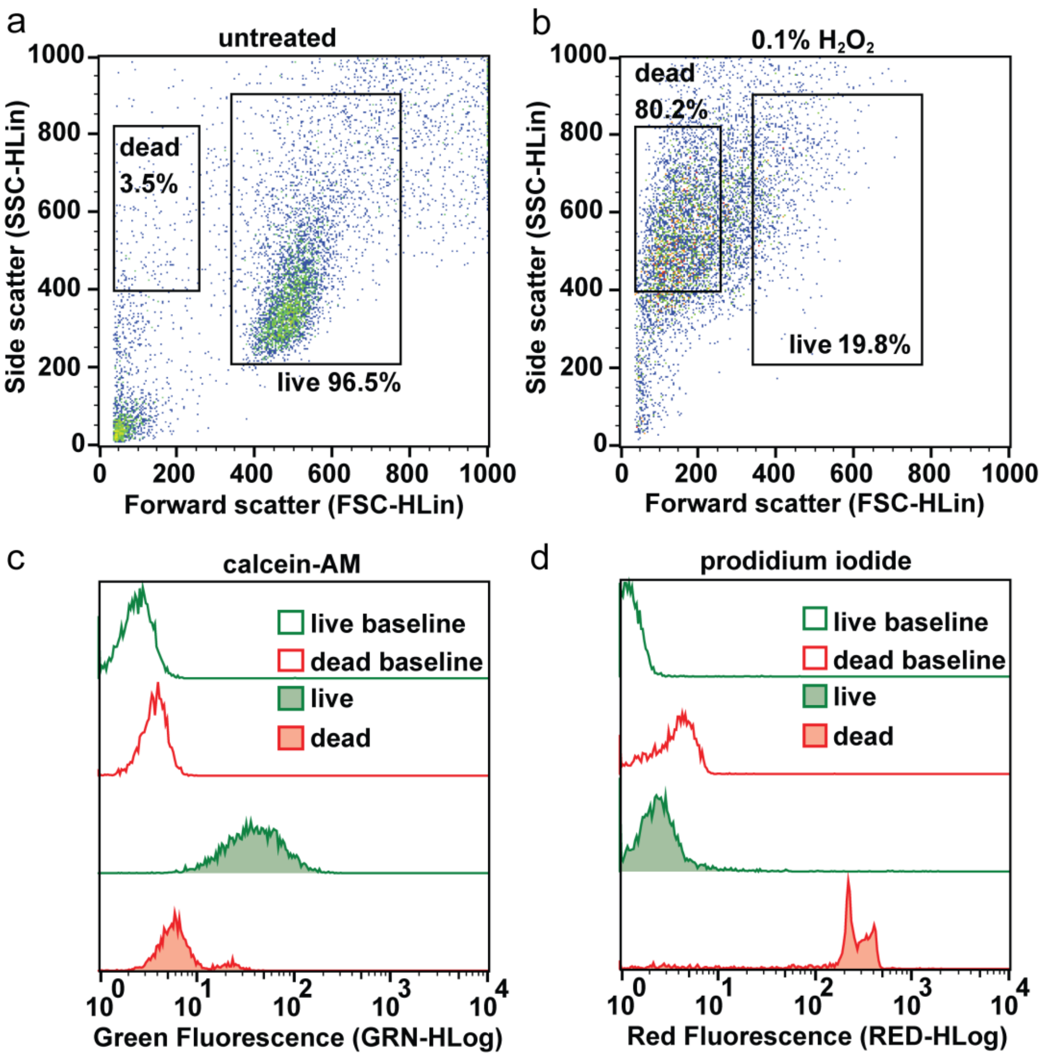

Supplementary Figure S3. *Localisation of live SH-SY5Y cells in flow cytometry FSC/SSC dot-plots. The top row shows (A) untreated cells and (B) cells treated with 0.1%* H_2O_2 *to obtain necrotic cells. The live and dead gates are depicted in the graphs. (C) Cells stained with calcein-AM counted in the live (green) and dead (red) gates respectively. The cells in the live gate display a clear increase in fluorescence intensity compared to baseline. (D) Cells stained with propidium iodide counted in the live (green) and dead (red) gates respectively. The cells in the dead gate display a clear increase in fluorescence intensity compared to baseline.*

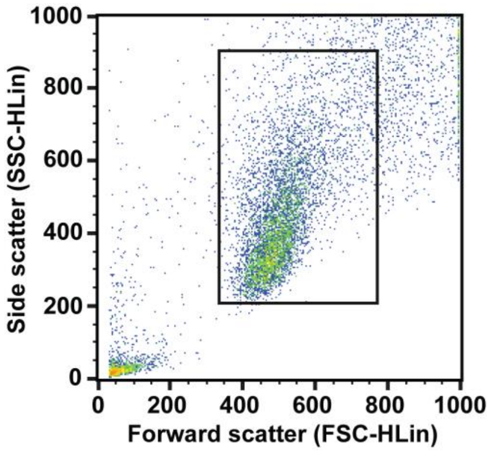

Supplementary Figure S4. *Flow cytometry FSC/SSC dot-plot of cells treated with 1 μM Aβ for 5 h. No shift in the cell population was observed and the majority of cells fall within the live gate.*

4. Optimisation of protocols for perturbation of endocytosis

The endocytosis inhibitors used in this study were toxic at high concentrations and/or prolonged incubation, therefore protocols needed to be optimized to find conditions where endocytosis was perturbed without concomitant cytotoxic effects. Optimisation was performed using the live/dead gates defined in Supplementary Fig. S3. Supplementary Fig. S5 shows FSC/SSC dot-plots corresponding to the treatment conditions applied for endocytosis perturbation (Fig. 2-5).

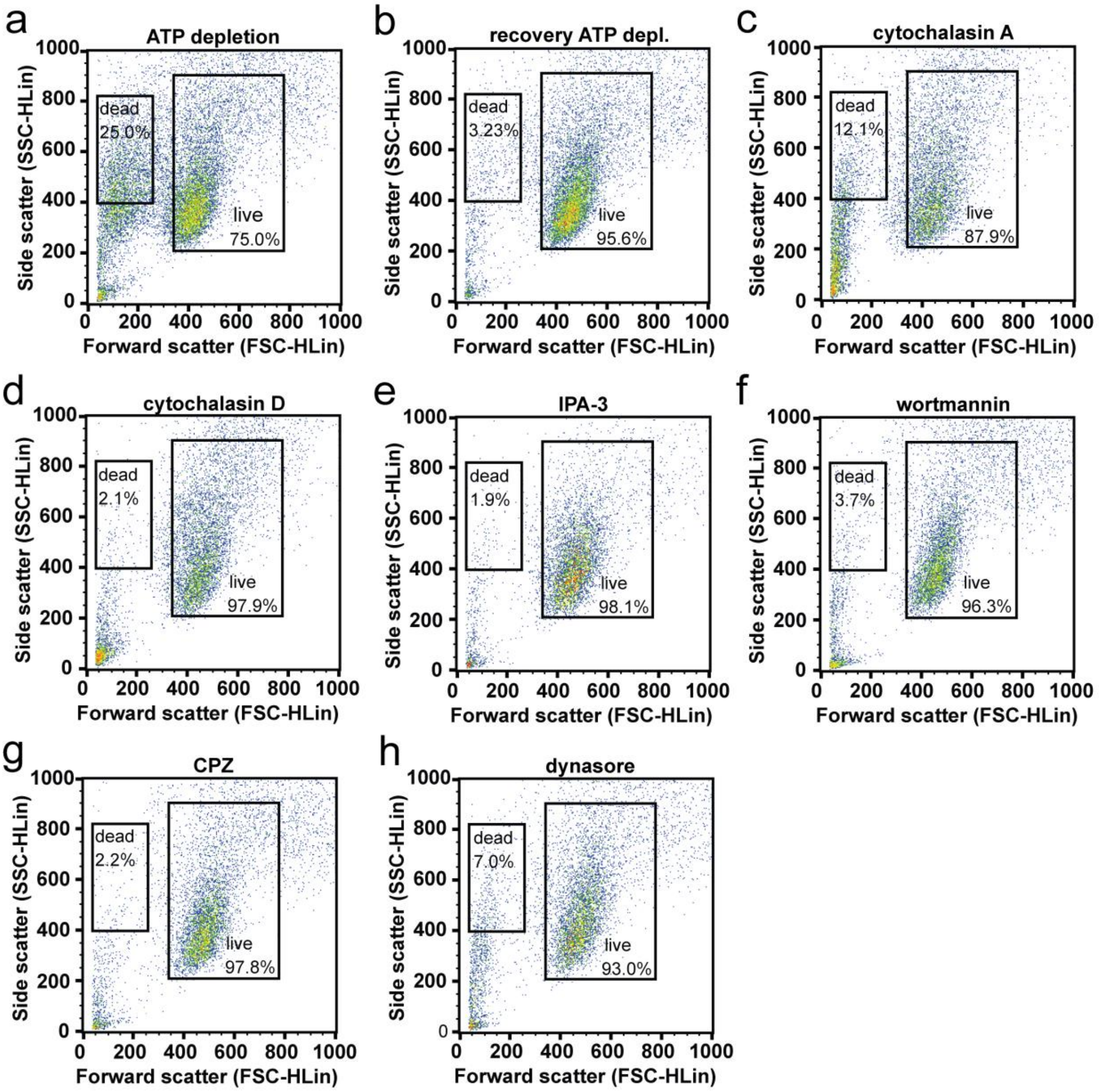

Supplementary Figure S5. *Flow cytometry FSC/SSC dot-plots of cell samples treated with the different endocytosis inhibitors used in this study. Data were recorded after the cells had been treated with inhibitors for the same duration of time as in the peptide incubation experiments. (A) ATP depletion solution 3 h, (B) ATP depletion 3 h followed by recovery 2 h, (C) cytochalasin A 10µM 90 min, (D) cytochalasin D 25µg/ml 90 min, (E) IPA-3 10 µM 90 min, (F) wortmannin 25nM 90 min, (G) CPZ 5 µg/ml 90 min and (H) dynasore 80 µM 90 min. Percentages denote the fraction of all gated events within the live and dead regions.*

5. Control for the transferrin pulse addition following endocytosis perturbation

The timespans of cell treatment with transferrin (Trf) (5 min) and Aβ (60 min) during endocytosis perturbation are different and effect the total time that cells are exposed to endocytosis inhibitors. We hypothesized that this may influence relative uptake as prolonged inhibitor exposure might lead to toxic effects. To test this, Trf was added in a 5 min pulse immediately after a 30 min pre-treatment period with each inhibitor, or following a 60 min pre-treatment as schematically depicted in Supplementary Fig. S6A. We compared Trf uptake in control samples (0.08% DMSO), in cells treated with the macropinocytosis inhibitor IPA-3 (Trf uptake should be unaffected) and in cells treated with dynamin inhibitor dynasore (Trf uptake should be affected) (Supplementary Fig. S6B). None or only minor effects could be observed, suggesting that toxicity due to the inhibitor does not develop during the 60 min incubation time window used for peptide incubations in this study. It was hence concluded Trf can be used as control for inhibition specificity, regardless of the pre-treatment duration.

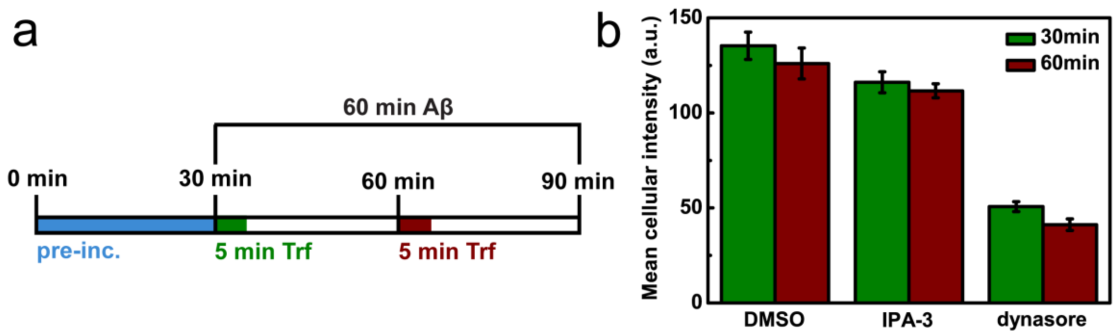

Supplementary Figure S6. *Experimental setup (A) and results (B) from flow cytometry quantification of the cellular uptake of Trf (5 min incubation) following 30 min or 60 min pre-incubation in 0.08% DMSO (control), 10 µM IPA-3 or 80 µM dynasore. Uptake is reported as the average fluorescence intensity (±SD) of the total number of gated live cells and baseline corrected for cellular autofluorescence. These pulse experiments were performed in triplicate (n=3).*

6. Optimisation of CPZ concentration and inhibition of clathrin mediated endocytosis including control samples

Since CPZ was found to be rapidly degraded in aqueous solutions, we optimised the concentration of this inhibitor by adding the Trf 5-min-pulse to cells which had already been treated with Aβ for 1 h, and thereafter followed the protocol for Trf treated cells as described in the Methods section. In doing so, we avoided the risk of the inhibitor being degraded during the experiment day. The results are depicted in Supplementary Fig. S7. Based on the plateauing effect in the reduction of Trf uptake, a concentration of 5 µg/ml CPZ was chosen for further experiments.

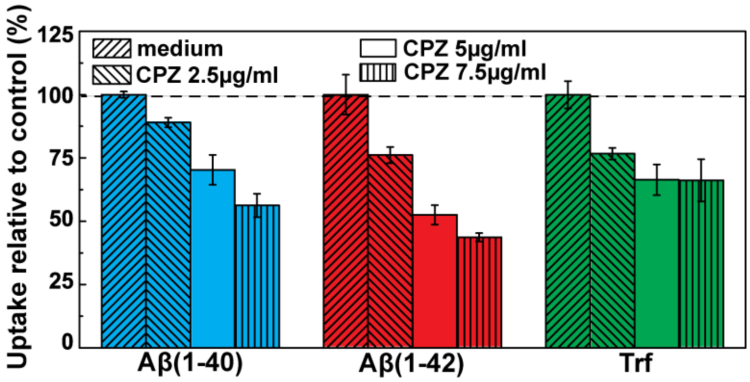

Supplementary Figure S7. *Uptake of Aβ (1 h incubation) and Trf (5 min incubation in Aβ pre-treated cells) with increasing concentrations of CPZ. Uptake is reported as the average fluorescence intensity (±SD) of the total number of gated live cells and baseline corrected for cellular autofluorescence. These experiments were performed in triplicate (n=3).*
